# Aspartame: Soffritti Responds

**Published:** 2007-01

**Authors:** Morando Soffritti

**Affiliations:** European Foundation of Oncology and Environmental Sciences “B. Ramazzini” Cesare Maltoni Cancer Research Center Bologna, Italy, E-mail: crcfr@ramazzini.it

As communicated in his letter, Abegaz represents Ajinomoto Corporate Services LLC. Ajinomoto, which holds 45% of the market share for worldwide aspartame production (Ajinomoto 2006), is well known for its aggressive and effective defense of its commercial interests. The action by Abegaz to reproduce portions of the opinion issued by the European Food Safety Authority (2006) regarding the results of our long-term carcinogenesis bioassay on aspartame (Soffritti et al. 2006) is clearly specious.
